# Investigation of sniffer technique on remote measurement of ship emissions: A case study in Shanghai, China

**DOI:** 10.1371/journal.pone.0274236

**Published:** 2022-09-16

**Authors:** Xiaobo Li, Ke Li, Qingpeng Ji, Feixiang Shen, Qiang Wu, Qiuyan Chen, Liangjing Luo, Xijia Bian, Wei Chen, Diming Lou

**Affiliations:** 1 Tongji University, Shanghai, China; 2 Shanghai Marine Diesel Engine Research Institute, Shanghai, China; 3 National Engineering Laboratory for Marine and Ocean Engineering Power System, Shanghai, China; Accra Technical University, GHANA

## Abstract

Shipping emissions have aroused wide concern in the world. In order to promote the implementation of emission regulations, this study develop a ship based sniffing technique to perform remote measurement of the SO_2_, NO_*x*_ and CO_2_ from ships entering and leaving Shanghai port at the open sea. The ship emission prediction model, Smoke diffusion model and source identification model were developed to automatically analyze the emission data and screen the object ship source based on Automatic Identification System (AIS) system. The fueling documents of the detected ship were obtained from maritime sector and the results precision of the sniffer technique was evaluated by comparing the measured Fuel sulfur content (FSC) with actual value deduced from fueling documents. The influences of wind speed and direction, object ship parameters and monitoring distance on the identification of object ship and accuracy of the calculated FSC were thoroughly investigated and the corresponding correction factors under different conditions were deduced. The modified emission factor ratio of CO_2_ to NOx were proposed in order to improve the accuracy. It is demonstrated that with wind speed higher than 2 m/s and test distance shorter than 400m, the sniffer technique exhibit high efficiency and accuracy for the remote emissions measurement of ship upwind with detection rate higher than 90% and test error of FSC below 15%. To reduce the influence of the wind direction, at least two sniffer systems were required to guarantee that at least one station is in the downwind of the ship lane. Based on the results and discussion, a novel sniffer monitoring system with two buoy based sniffing stations placed close to each side of the ship lane far off shore was proposed to realize the remote monitoring of ship emissions.

## 1. Introduction

Shipping businesses occupy 80% to 90% of global transportation and make important contributions to the world economy. However, in times of increased environmental awareness, the atmospheric pollution caused by ships has aroused wide concern in the world. Atmospheric pollution due to ships is one of the main issues within harbour management due to the degradation of local air quality, adverse health effects, and climate implications. It has been proved in many researches that CO_2_, SO_2_ and NO_*x*_ emitted by ships account for 2.7%, 4%-9% and 15% [1, 2] of all anthropogenic pollution, respectively. In order to reduce adverse health effects of shipping exhaust and improve air quality, In 1997, the International Maritime Organization(IMO) formulated the International Convention on Pollution Prevention MARPOL 73/78 Annex VI—“Regulations for Preventing Air Pollution from Ships”. Technical Code of Annex VI Regulation 13 has an achievement to a comprehensive level of Tier III in detaining the NOx emission from ship propulsion and electrical power plant. And the sulfur content of marine fuel oil worldwide should not exceed 3.5% since 2012, and the sulfur content should not exceed 0.5% after 2020 [3].

In order to reduce the impact of ship exhaust on the air quality of coastal cities, countries in Europe and America set stricter standards demanding the ships passing through emission control area (ECA) use low sulfur oil or other equivalent desulfurization equipment. Since 2015, in Emission control areas such as the Baltic Sea, the North Sea, the English Channel and the coastal waters of North America, diesel fuel with a sulfur content not exceeding 0.1% is required for the ship. In China, emission control areas in Pearl River Delta, Yangtze River Delta, and Bohai Rim (Beijing-Tianjin-Hebei) are officially established at 2016 and it is required that fuel oil of ships sailing or berthing in the control areas should exhibit a sulfur content of not more than 0.5% m/m since January 1, 2019, and limit is further reduced to 0.1 m/m for berthing ships since January 1, 2020 [4].

Due to the large price difference among diesel fuels of different sulfur content [5], it is not realistic to expect the ship owners to use high-cost-low-sulfur diesel fuel consciously. The supervision of maritime sector is important for the enforcement of the emission regulations. However, conventional inspection approach such as checking the oil change records in the logbook and oil sample detection are far from fulfilling the practical requirement since it is difficult to board the ship and the inspection efficiency is very low. Thus there is an urgent need for non-boarding, remote and fast ship fuel sulfur content detection technology to realize efficient and precise supervision of the ship emission. Western developed countries have more than ten years of experience in the management of ship emission control areas and the accumulated research findings shows that the sniffing method is currently the mainstream telemetry method for sulfur content detection in marine fuel oil [6].

Chalmers University in Sweden took the lead in launching the identification of the Gross Pollsing Ships (IGPS) project to study the application of sniffing methods on the monitor of atmospheric pollutants from ship exhaust and identify vessels that illegally use heavy sulfur oil in the Rotterdam port area [7]. They developed the aircraft as the carrier of sniffing sensor, which will initially identified the suspected ship at a height of 200 to 300 m (concentration error 40%) with differential absorption spectroscopy method, and then descended the flight altitude through the ship’s exhaust zone to further verify the sulfur content (the error is about 20% when the sulfur content is 1%), and the maritime administration is notified to board the ship to make the final check [8, 9]. The Finnish Institute of Meteorology with a number of colleges and universities carried out the Shipment-Induced NO_*x*_ and SO_*x*_ Emissions-Operational Monitoring Network (SNOOP) project and apply sniffing technology to monitor the oil sulfur content of marine passing in the channels of the port of Helsinki and Turku. The results show that ships using tail gas treatment technologies such as direct water injection or air humidification emit very little SO_2_, which is equivalent to using low-sulfur oil [10]. In September 2009, the EU Joint Research Center JRC applied a variety of methods for remote monitoring of ship exhaust NOx and SO_2_. The results demonstrate that the sniffer method is the most effective technology with highest accuracy. The measurement error of the sniffer located on the shore, board and aircraft is about 0.23% (m/m), 0.15% (m/m), 0.18% (m/m), respectively [6], indicating that the accuracy of the sniffer is highly influenced by the test distance. The EU Joint Research Center carried out the experiments in the Gulf of Genoa with aircraft-based monitoring equipment (electrochemical method). The emission exhaust of 10 ships was monitored and the results indicate that the aircraft-based sniffer method has better signal-to-noise ratio due to close contact with the exhaust [11]. However, this method require that the sample is taken inside of the plume, i. e. the inlet has to be located high enough or the aircraft has to fly low enough, respectively, to match the plume height. The Bremen University applied sniffer and differential absorption spectroscopy technology to remotely monitor the oil sulfur content of the ship passing through the port of Hamburg and the estuary [12]. It is found that the accuracy of the monitoring points along the channel is significantly better than the estuary lighthouse, which is due to the fact that the former is closer to the ship.

To summarize, the previous researches have proved that the sniffer method is a feasible way for the detection of ship emissions, and the precision is highly dependent on the test distance. Actually, the ground based sniffer station is not a realistic method due to the fact that most ships near the port will shut down the main engine and the detected data cannot reflect the actual sulfur content. The air based sniffer station requires skilled driving abilities of pilot and seems unable to satisfy the automation monitoring requests. In addition to the test distance, the wind velocity and direction as well as the object ship parameters also exert an important influence on the result uncertainties since the diffusion factors of NO_*x*_, CO_2_ and SO_2_ are related with multiple gas phase parameters. However, the influences of these factors were not thoroughly analyzed qualitatively and the algorithms on how to screen the detected ship automatically and accurately from amounts of ships through the monitoring site in the same time interval are still under investigated.

In this study, ship based sniffer technology was applied on pollutant emission monitoring in Shanghai WuSong port with a heavy traffic of 5000 ships per day. The meteorological parameters were monitored online with weather station and the trajectory of ship was collected through Automatic Identification System (AIS) system. On the basis of these data, The emission prediction model, Smoke diffusion model and source identification model were developed and optimized to screen the target ship and the influences of wind speed and direction, ship parameters and test distance on the test accuracy were thoroughly investigated and finally the principle of location of sniffer equipment was proposed.

## 2. Measurement and model approaches

### 2.1 Integrated sniffer station

**Fig 1:**
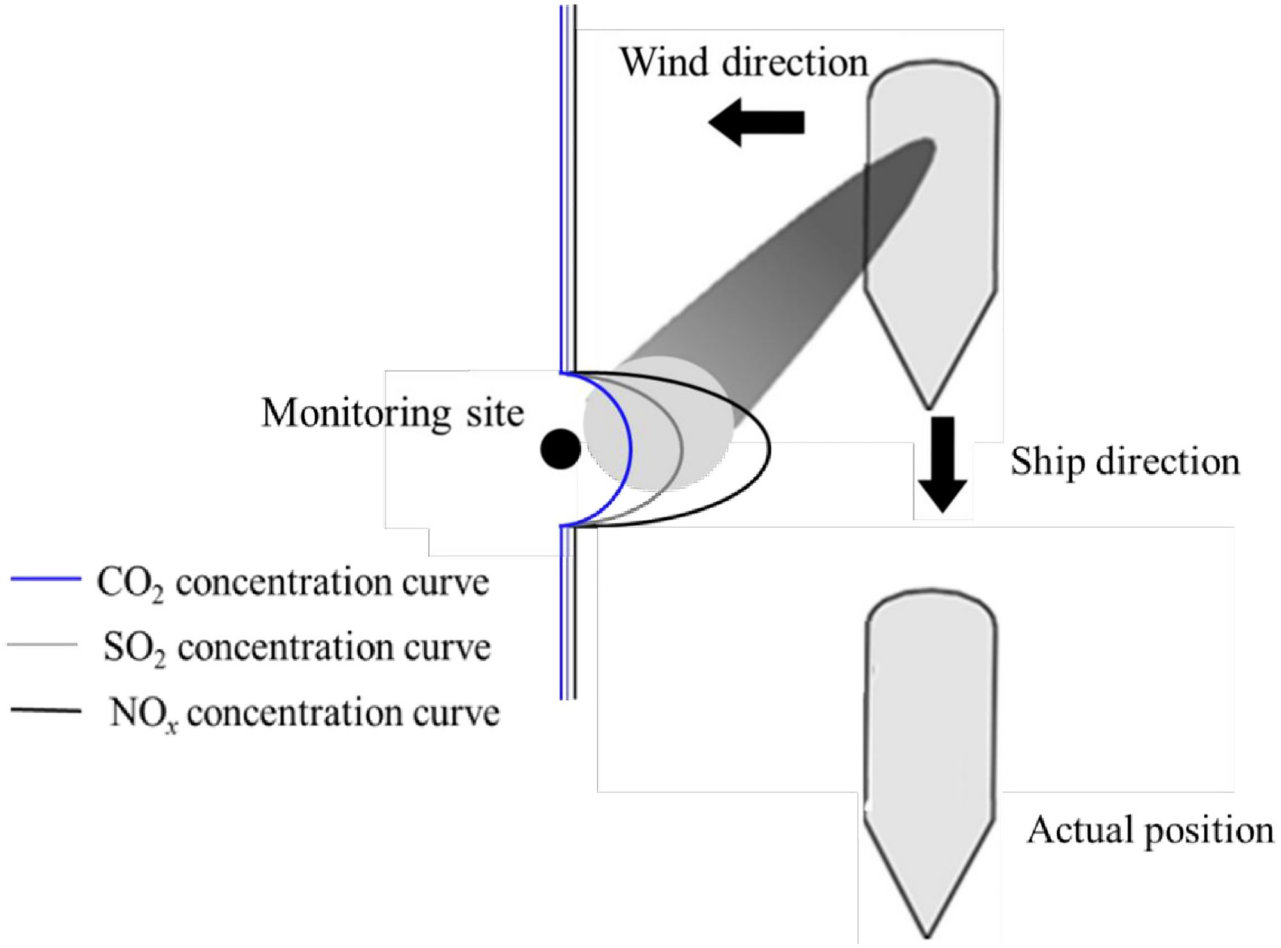
The schematic and the profile of the sniffer station.

The integrated sniffer station is composed of NO_*x*_ analyzer (Thermo Model 42i), SO_2_ analyzer (Thermo Model 43i), CO_2_ analyzer (Thermo Model 410i) and multifunctional meteorological instrument. The measure principle of NO_*x*_, SO_2_ and CO_2_ analyzer is respectively based on Chemiluminescence, UV fluorescence and Infrared absorption, and the corresponding instrument deviation is ±1 ppb, ±1 ppb and ±1 ppm, respectively. The meteorological instrument is mounted at the inlet of the analyzer to record the temperature, humidity, wind direction and wind speed at the monitoring site. The schematic and profile of the sniffer station is shown in Fig 1.

**Fig 2:**
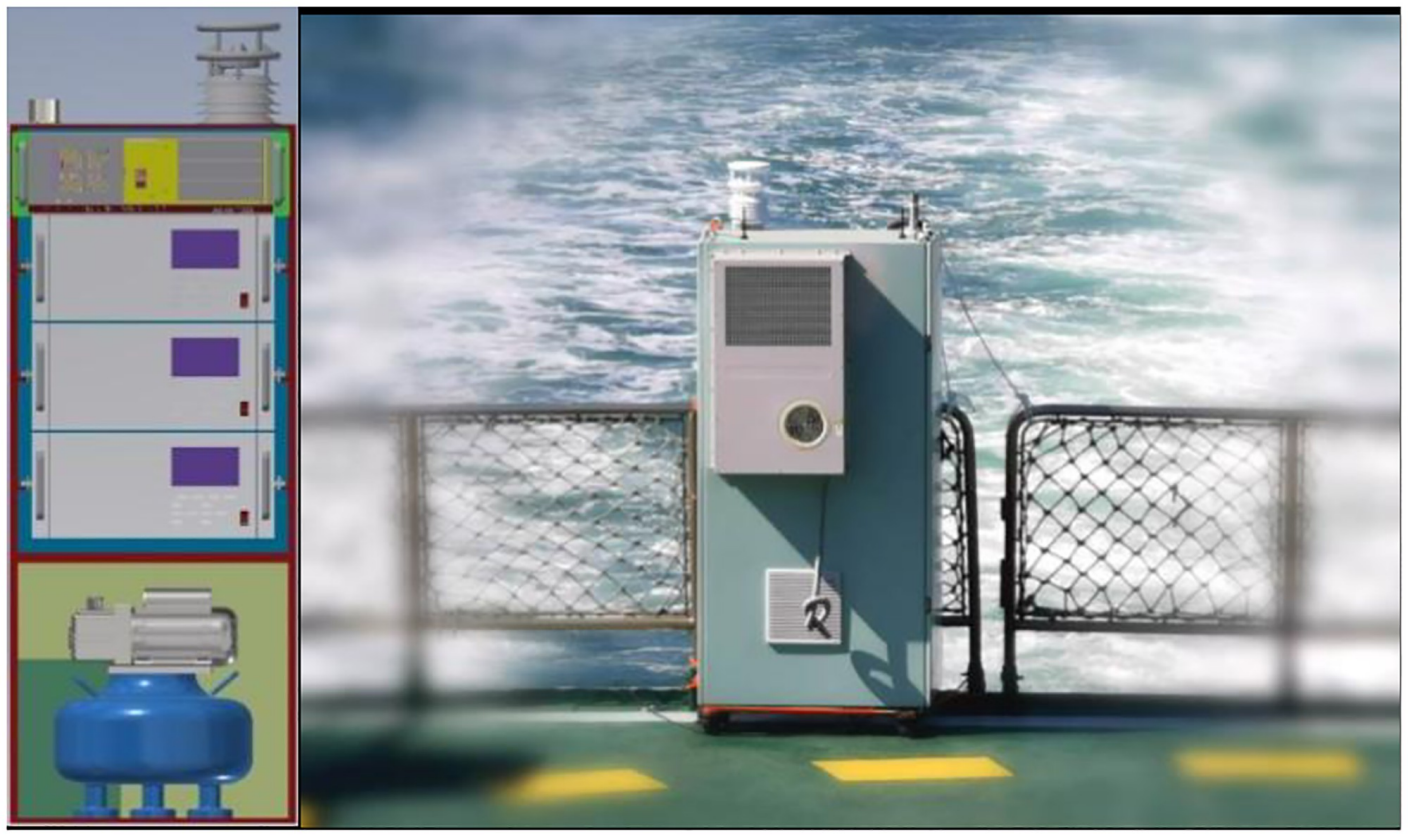
Schematic theory of sniffing method(the shadow represent the distribution of the ship plumes).

The sniffer method is based on three assumptions [13]: one is that the carbon ratio in the fuel oil of different sulfur content are all assumed to be around 87%; This assumption is supported by oil testing data that C and H elements account for more than 95% in the oil [14–16]. The sulphur content varies from 0.1% to 1% in oil obtained from different extraction methods. Thus the sulfur content exert no influence on the carbon ratio. The second assumption is that most C and S element in the oil will exist in the form of CO_2_ and SO_2_ after combustion of the oil with oxygen, the proportion of other carbonic oxide and sulfate is so small that can be neglected. Based on the material balance formula, the carbon oxidation rate in the liquid fuel is basically not less than 98%, and SO_2_ accounts for more than 95% of the sulfur oxides in the combustion products [17, 18]. The third assumption is that the CO_2_ and SO_2_ diffusion along wind direction is proportional to the distance, leading to a constant ratio of SO_2_ to CO_2_. The difference in sedimentation rate caused by the variation of molecular weight is negligible in the diffusion process. Based on the dry deposition research of atmospheric components, the dry deposition is usually manipulated by large space-time scale meteorological factor [19]. Thus there is no need to take this phenomenon into consideration since the time range of diffusion process is within an hour. Besides, although SO_2_ will be converted to sulfuric acid at a rate of 20% to 30% per hour, the loss is still very small in the first few minutes. Based on the above assumptions, the signal fluctuation can be detected at downwind of the ship exhaust, as illustrated in Fig 2. And the concentration of the pollutant can be calculated through the following equation:

$${\text{SO}}_{\text{2}}{\text{[ppb]=SO}}_{\text{2e}}{\text{[ppb]-SO}}_{\text{2b}}\text{[ppb]}$$ (1)

$${\text{CO}}_{\text{2}}{\text{[ppm]=CO}}_{\text{2e}}{\text{[ppm]-CO}}_{\text{2b}}\text{[ppm]}$$ (2)

Where SO_2e_ and CO_2e_ is the concentration of SO_2_ and CO_2_ detected by the sniffing sensors, SO_2b_ and CO_2b_ is the concentration of SO_2_ and CO_2_ in the background.

Then the sulfur content of the oil can be calculated as follows:

$$\text{SC}=\frac{{\text{S}}_{\text{oil}}\text{[kg]}}{{\text{C}}_{\text{oil}}\text{[kg]}}=0.87\times \frac{{\text{SO}}_{\text{2}}\text{[ppm]}\times \;32}{{\text{CO}}_{\text{2}}\text{[ppm]}\times 12}\;=\left(\text{0}.232\times \frac{{\text{SO}}_{\text{2}}\text{[ppb]}}{{\text{CO}}_{\text{2}}\text{[ppm]}}\right)\text{\%}$$ (3)

Where SC is the sulfur content of oil, S_oil_ and C_oil_ is the mass of S and C elements in oil. The method of using concentration peaks measured at high temporal resolution to investigate the contribution to air quality of a single ship moving in the ship lane was already used in previous research and the difference of the method used in this paper is to combine emission source identification with the detection of the pollutant.

### 2.2 Monitoring sites

The integrated sniffer station is located on the monitoring ship anchored at downwind of sea channel at Shanghai Port, The monitor distance is variable from 150 to 1000 m by changing berth location. The monitoring ship is not allowed to anchor too near to the ship channel since enough distance of above 400 meters between the ship and the channel is demanded to avoid any collision accident. So the monitoring ship will move around the channel and chase the object ship if a monitoring distance of shorter than 400 m is demanded. The monitoring site is far from the land in order to be free from additional pollution sources. The concentrations of SO_2_ and NO_*x*_ in the air background are low and stable. Since Shanghai port is one of the busiest port in China, the ship traffic is rather dense and nearly 5000 ships will pass through the monitor site one day, which is beneficial for fast data collection.

### 2.3 Emission source identification

Due to the heavy ship traffic, the emission source identification process is much challenging than the experiments in previous literature. When a considerable increase of NO_*x*_, SO_2_ and CO_2_ is detected by the integrated sniffer station, there are at least 5 ships within the effective monitoring range in most cases, the traditional emission source identification method can hardly recognize the only emission source. In this paper, a two-step source identification model is proposed and the detected NO_*x*_ concentration was applied as the driver of source identification procedure since the NO_*x*_ signal peak is much obvious than SO_2_ and CO_2_ due to a combined effect of ship emission pollutant distribution and the corresponding sensor minimum resolution of different sensor, as shown in Table 1.

**Table 1:** Emission distribution and sensor resolution of different gaseous pollutant.

Gaseous pollutant	Concentration in pipe	Minimum resolution of sensor
NO_*x*_(NO and NO_2_)	800–1200 ppm	1ppb
SO_2_	10–500 ppm	1ppb
CO_2_	50000-100000ppm	1ppm

In the first step, all the ships within monitoring range is suggested as suspect emission source and their information such as ship’s type, identification number, size, position, course and speed were all recorded according to the AIS(Automatic Identification System) database. Then the exhaust emission of NO_*x*_ can be calculated as follows:

$${\text{E}}_{\text{NOx}}=\sum_{\text{j}}{\text{P}}_{\text{j}}{\text{L}}_{\text{j}}{\text{T}}_{\text{j}}{\text{E}}_{\text{j}}$$ (4)

Where E_NO*x*_ is the exhaust emission of NO_*x*_, P is the propelling power of the ship, L_F_ is the ship load factor, T is the operating time under certain conditions, E is the emission factor of NO_*x*_, j represents different power equipment i.e the main engine and auxiliary engine. The P can be obtained from the AIS database, and the L_F_ of the main engine can be calculated through the following question:

$${\text{L}}_{\text{F}}={\left({\text{V}}_{\text{AS}}/{\text{V}}_{\text{MS}}\right)}^{3}$$ (5)

where V_AS_ is the real speed of the ship and V_MS_ is the maximum speed that are both available in the AIS database. Due to non-uniformity of AIS data on a time scale, the coherent-differential method [20] was applied to obtain the accurate V_AS_. If the V_MS_ is not available, the V_AS_/V_MS_ is set as the default value of 0.75. The L_F_ of auxiliary engine were listed in S1 Table in S1 File.

The emission factor E of different power equipment is summarized at S2 Table in S1 File according to the previous literature.

The Gauss diffusion model was applied to calculate the theoretical concentration of the NO_*x*_ at the monitoring site diffused from the ship pipe. And the equation is as follows:

$$\text{C(}x\text{,}y\text{,}z\text{,}H\text{)}=\frac{E_{\text{NOx}}}{\text{2}\pi u\sigma_{y}\sigma_{z}}\text{exp(}\frac{-y^{\text{2}}}{{2\sigma }_{y}^{\text{2}}}\text{)}\left\{\text{exp}\left[\frac{\text{-(}z{\text{-}H\text{)}}^{\text{2}}}{{2\sigma }_{z}^{\text{2}}}\right]\text{+exp}\left[\frac{\text{-(}z{\text{+H)}}^{\text{2}}}{{2\sigma }_{z}^{\text{2}}}\right]\right\}$$ (6)

Where u is the average wind velocity. *x*, *y* and z are the distance between target ship and monitoring site at different dimension. *H* is the height of the ship pipe above the ground monitoring equipment. *σ*_*y*_ and *σ*_*z*_ is the diffusion coefficient in the *x* and *y* direction, respectively. The diffusion parameters are the characteristic quantities that represent the diffusion range and rate, i.e the standard deviation of the normal distribution function. In order to determine these diffusion parameters more precisely, various diffusion parameter estimation methods based on experimental conditions are proposed combining the concentration field and meteorological parameters. Among them, the most popular method is the P-G diffusion curve proposed by Pasquill and Gifford [21]. The suggest that diffusion parameter σ obeys the following rules: (1) σ increases with distance from monitoring site to the source; (2) the value of σ in an unstable atmosphere condition is greater, and the stronger of atmospheric turbulence movement, the greater of σ value; (3) the σ value on rough ground is greater than that on flat ground. According to the Gaussian dispersion model, when the emission source is movable, the velocity of emission source will contribute little to the dispersion of the gaseous pollutant since the mass of the gaseous pollutant is so small that the initial velocity caused by inertia can be ignored. The whole calculation process is as follows: Firstly, the local conventional meteorological observation data were obtained from the multifunctional meteorological instrument, then the atmospheric stability level can be evaluated according to Pasquier method and finally the diffusion parameter σ can be determined through the following equation:

$$\sigma_{y}=\gamma_{\text{1}}x^{\alpha_{\text{1}}}$$ (7)

$$\sigma_{z}=\gamma_{2}x^{\alpha_{2}}$$ (8)

Where *x* is the component of distance between ship and monitoring site in the wind direction, *γ*_1_ and *α*_2_ is the empirical parameters related to the atmospheric stability level and *x*. And they can be obtained from document GB3840-91 proposed by National Environmental Protection Bureau of China.

When a NO_*x*_ signal fluctuation emerged, the routes of each suspect ship in last 20 minutes were divided into many discrete points with a fixed interval of 10 meters. The theoretical NO_*x*_ concentrations diffused from the suspect ship to the monitoring site in each discrete position was calculated through the above ship emission prediction model and Gauss diffusion model. The position with the max theoretical NO_*x*_ value was labeled as the “Special position”. The maximum of these theoretical concentrations was compared with the measured value. If the difference is not within an acceptable range, the ship is removed out of the “suspect ship list”.

**Fig 3:**
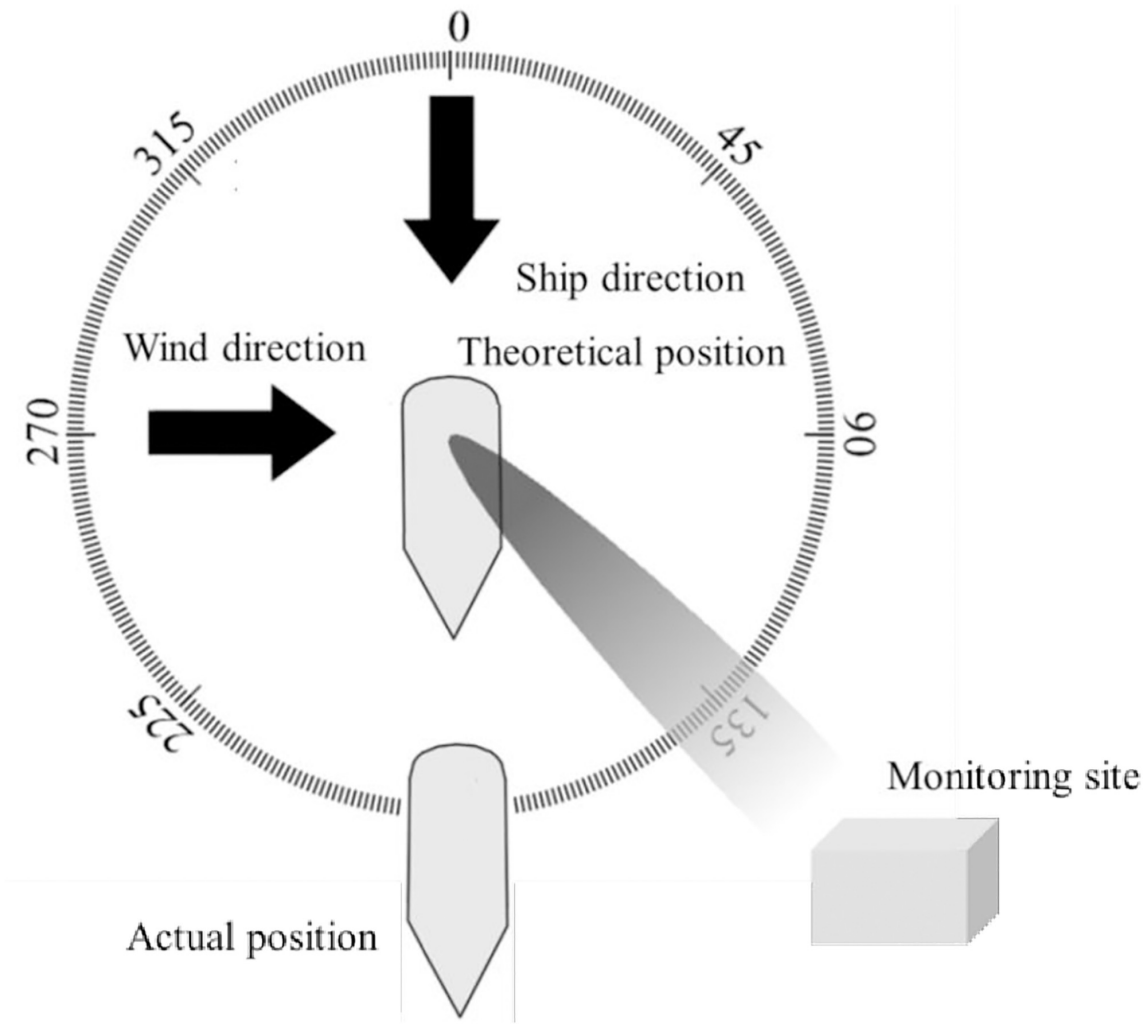
Schematic principle of the second source identification process(the shadow represent the distribution of the ship plumes).

After the first emission source identification procedure, if only one ship remain on the “suspect ship list”, it is considered as the target ship. Otherwise, the second emission source identification procedure is necessary to further determine the target ship. The process is shown in Fig 3. Based on the wind direction and the ship course, the diffusion direction of the NO_*x*_ of each ship can be obtained through vector superposition. With the recorded time of signal fluctuation emergence, the theoretical source position of the NO_*x*_ responsible for the signal peak can be clarified. The difference between theoretical source position and “Special position” (abbreviated as position error) is deemed as the judgement criterion. The ship with lowest position error can be considered as the target ship. If two or more ship’s position error are too close to distinguish the lowest value, which will occurred due to the short distance between ships, no ship will be targeted, indicating the failure of emission source identification in this case.

### 2.4 System feasibility evaluation

The following evaluation indexes were used to evaluate the feasibility of ship exhaust pollutant remote monitoring system:

1. Fluctuation Generation Rate, defined as the ratio of number of pollutant signal fluctuation to the ships that passed through the monitoring site, was applied to evaluate the remote sensing range of the integrated sniffer station.
2. Fluctuation Recognition Rate, defined as the ratio of fluctuation number recognized by the system automatically to the actual fluctuation number, was applied to evaluate the background deviation of the signal on the sea and the intelligence of the monitoring system. If the background value of the NO_*x*_ signal drift significantly, a considerable amount of fluctuation would not be recognized precisely.
3. Diffusion Model Accuracy, defined as the ratio of theoretical concentration of the pollutant diffusion to actual concentration tested by the sniffer station, was applied to evaluate the accuracy of the pollutant Diffusion model and the pollutant prediction model. In the case that the target ship is recognized and marked with no doubt, the model accuracy can be calculated and the influence of wind direction, wind speed on the model accuracy can be intensely studied.
4. Source Trace Accuracy, defined as the difference between “Special position” and theoretical source position, was applied to evaluate the accuracy of the source trace in the second Emission source identification. In the case that the target ship is recognized and marked with no doubt, the Source Trace Accuracy can be calculated and the influence of wind direction, wind speed on the source trace process can be intensely studied.
5. Ship Marking Rate, defined as the ratio of number of marked detected ships to number of all ships passing through the monitoring site. It is a comprehensive performance index based on Fluctuation Generation Rate, Fluctuation Recognition Rate, Model Accuracy and Source Trace Accuracy, reflecting the feasibility of the sniffer technique in the field of ship exhaust remote monitor.
6. Valid Ship Marking Rate, defined as the ratio of the quantity of marked ships whose oil content can be calculated through equation (2–1) to the number of all ships passing through the monitoring site. Not all the marked ship’s oil content can be obtained since the CO_2_ signal fluctuation may not be detected due to the relatively poor accuracy of CO_2_ sniffer equipment with the minimum unit of ppm.
7. Accuracy of the oil sulfur content, defined as the difference between calculated sulfur content and real sulfur content obtained from the oil document provided by the marine bureau. Actually, the FSC from fueling documents is not credible and the true FSC must come from a direct test of the fuel. According to our collected dozens of fuel test data and maritime law enforcement practice, the difference between the fuel direct test data and the result from fueling documents may mainly be caused by the change of the ship fuel in order to reduce fuel costs. Many bulk cargo ships will change fuel while most large container ships from eminent company such as COSCO and MSC tends not do this and results from their fueling documents is more credible. Thus most data used in this manuscript were obtained from large container ships.

A detailed case calculation is illustrated in S3 Table in S1 File.

## 3. Results and discussion

### 3.1 Fluctuation generation rate

To investigate fluctuation generation rate of the sniffer station near the ship channel, the statistical data of the 3-mont experiments were intensely analyzed and the results were classified by monitoring distance as well as wind speed and direction.

**Fig 4:**
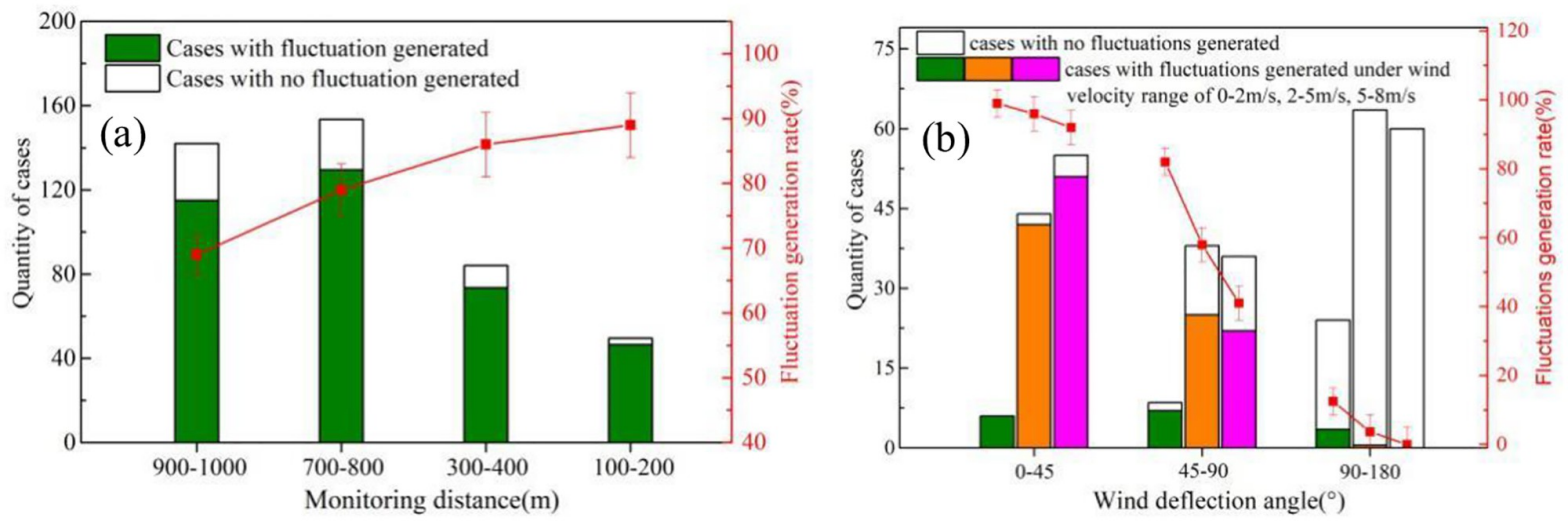
Results of fluctuation generation under (a) different monitoring distance; (b) different wind deflection angle and velocity with monitoring distance of 400m.

Fig 4(a) illustrates the average quantity of ships and fluctuations in a test day at different monitoring sites, thus the relation between fluctuation generation rate and monitoring distance can be obtained. It can be seen from the results that with the decrease of monitoring distance, the fluctuation generation rate exhibit an obvious rise. With monitoring distance higher than 900 meters, the fluctuation generation rate is below 70%, indicating that more than 30% of the ships through the monitoring site will not leave any exhaust traces due to low initial concentration and high disperse dilution. Within a distance range of 400m, the fluctuation rate achieved higher than 85%, and most escaped ones were small vessels with extremely low exhaust flows and not necessarily suggested as the targets of emission regulation.

Fig 4(b) shows the influence of wind speed and direction on the fluctuation generation rate at the monitoring distance of 400m. With the increase of wind speed, less fluctuation can be detected since the disperse dilution of the pollutant is in positive correlation with the wind speed according to Gauss diffusion model. In order to further quantify the influence of wind direction, the wind deflection angle was defined as the angular separation between vector of wind direction and the vector from object to monitoring site. Thus the wind deflection angle is ranging from 0°-180°. The range of 0°-90° and 90°-180° represent the downwind and upwind location of the monitoring site, respectively. With wind deflection angle higher than 90°, hardly any fluctuation can be detected since the wind tends to carry the exhaust pollutant to the other side. In such a case, several fluctuations can still be detected due to the pollutant free diffusion velocity. With wind deflection angle lower than 45°, the fluctuation generation rate is near 100%, indicating that 4 sniffer stations are demanded around the ship lane to guarantee high monitoring efficiency.

### 3.2 Fluctuation recognition rate

**Fig 5:**
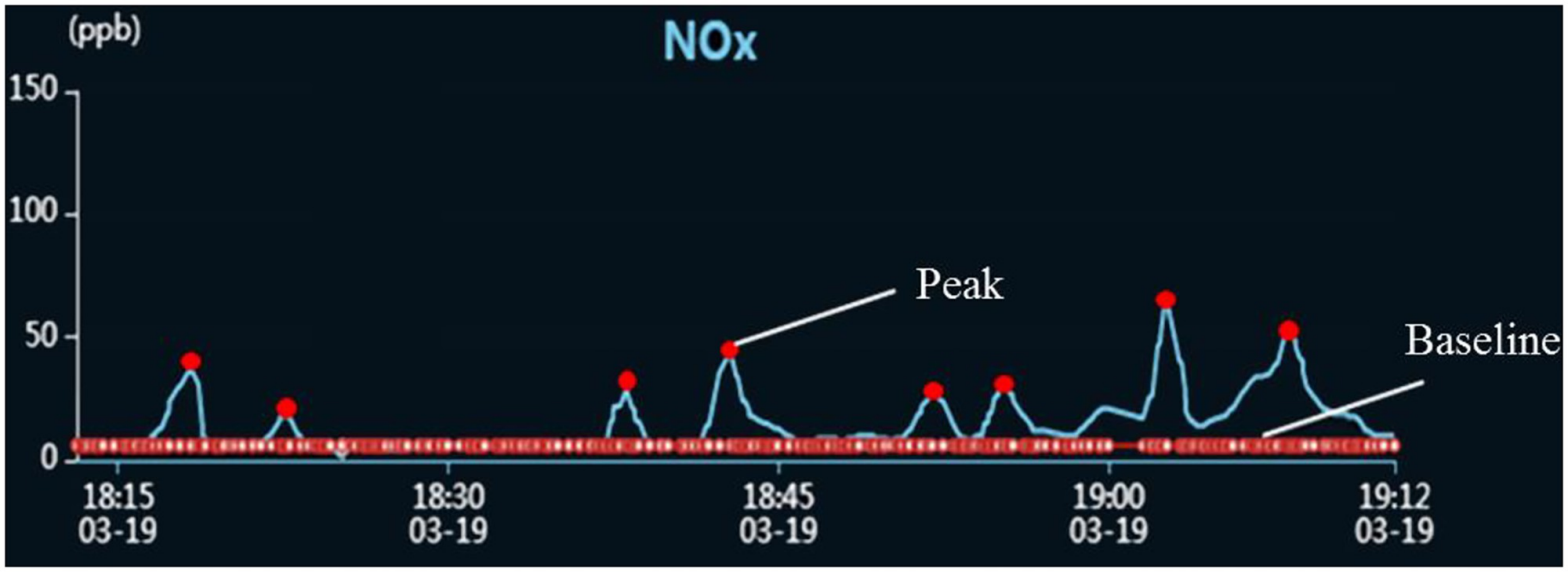
NO_*x*_ signal with smooth baseline.

**Fig 6:**
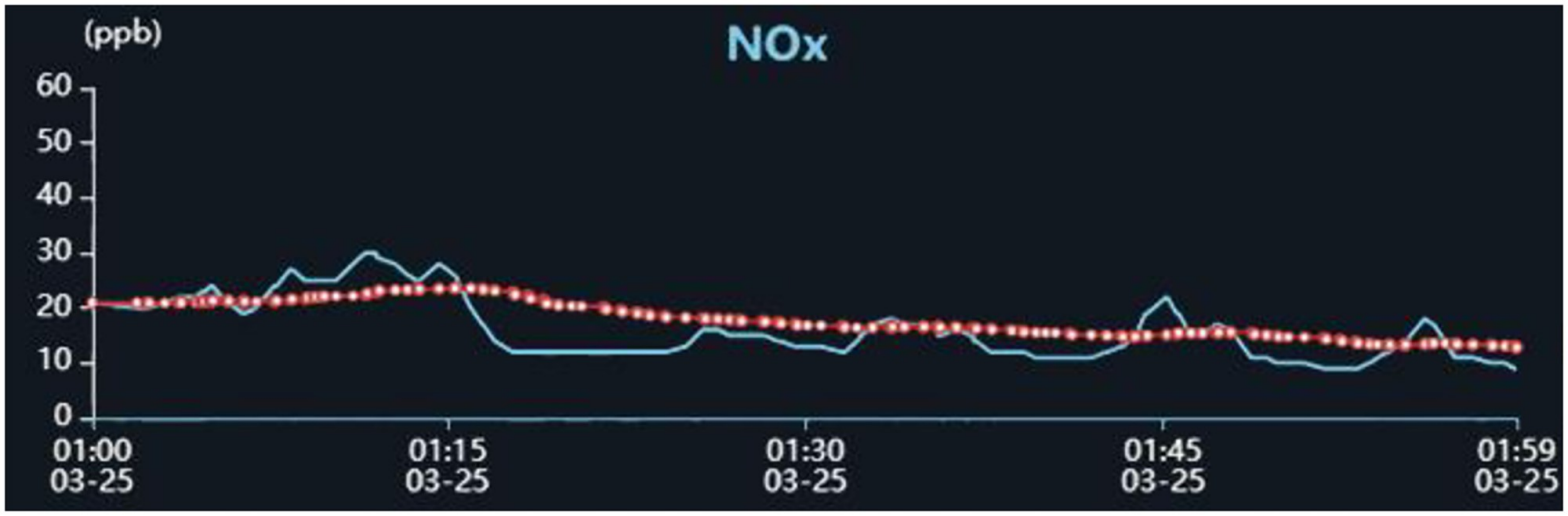
NO_*x*_ signal with drift baseline.

**Fig 7:**
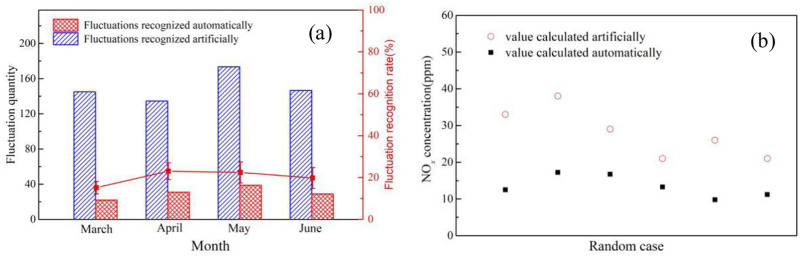
Comparison of (a) quantity of fluctuations recognized automatically and artificially; (b)NO_*x*_ concentration value calculated artificially and automatically.

Once the signal fluctuation was generated, it is easy to recognize the fluctuation and calculate the detected pollutant concentration artificially through Eqs (1) and (2). In order to realize complete automation of the whole detection process, the conventional automatic recognition algorithm was applied to carry out the fluctuation recognition automatically through distinguishing the baseline and peak point of the pollutant signal, as illustrated in Fig 5. However, due to the high humidity and meteorological changes offshore, the frequent baseline drift will exert a significant influence on the fluctuation recognition rate and the accuracy of the calculated pollutant concentration, as shown in Fig 6. Thus the conventional fluctuation recognition method seems not feasible in the ship pollutant remote detection. It can be seen from Fig 7(a) and 7(b) that the recognition rate is rather low and the calculated pollutant concentration automatically exhibit an obvious difference from the value obtained artificially.

**Fig 8:**
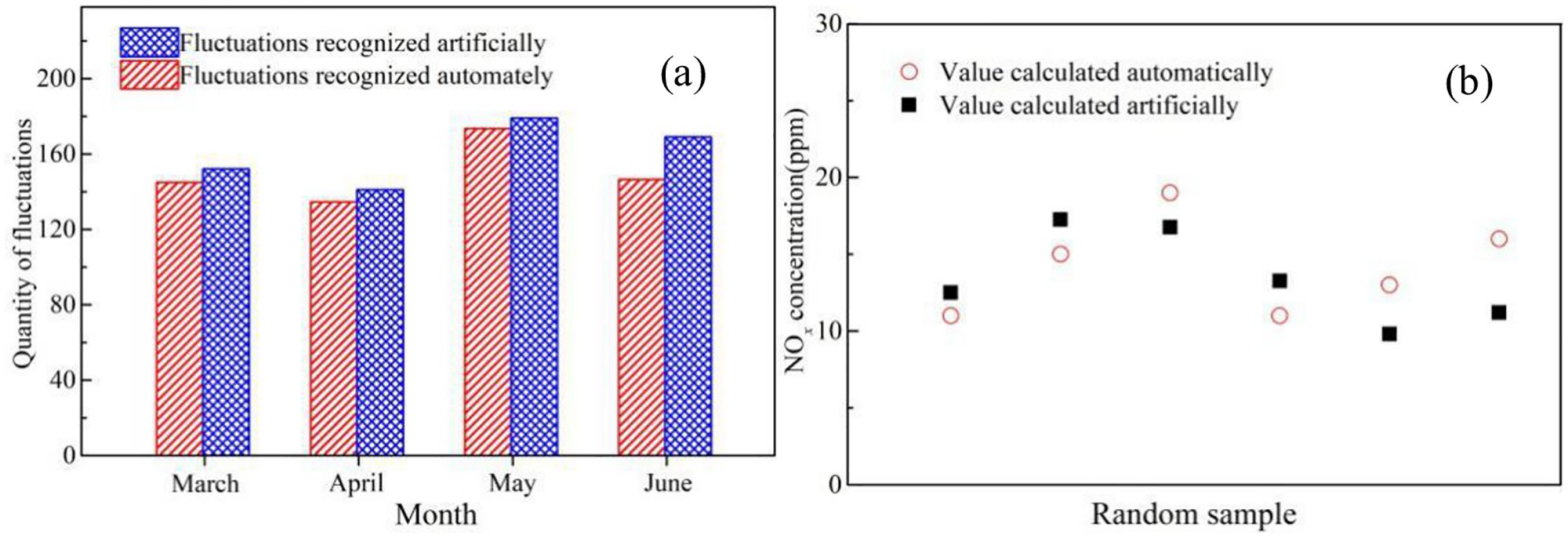
Comparison of (a) fluctuation quantities recognized automatically and artificially; (b)NO_*x*_ concentration value calculated artificially and automatically.

In order to minimize the influence of the baseline drift on fluctuation recognition and accuracy of the calculated pollutant concentration, a dynamic baseline drift method was applied to calculate the baseline function online through differential calculation method by recognizing the rising and falling edges of the signal, as shown in S1 Fig in S1 File. The pollutant concentration was then obtained by reducing the corresponding base value from the peak value. Fig 8 illustrates the results based on the new method. The average fluctuation recognition rate reached near 95% and the accuracy of the calculated pollutant concentration was also as high as 90%, which can satisfy the demand of remote monitoring over a complex marine environment.

### 3.3 Model accuracy

**Fig 9:**
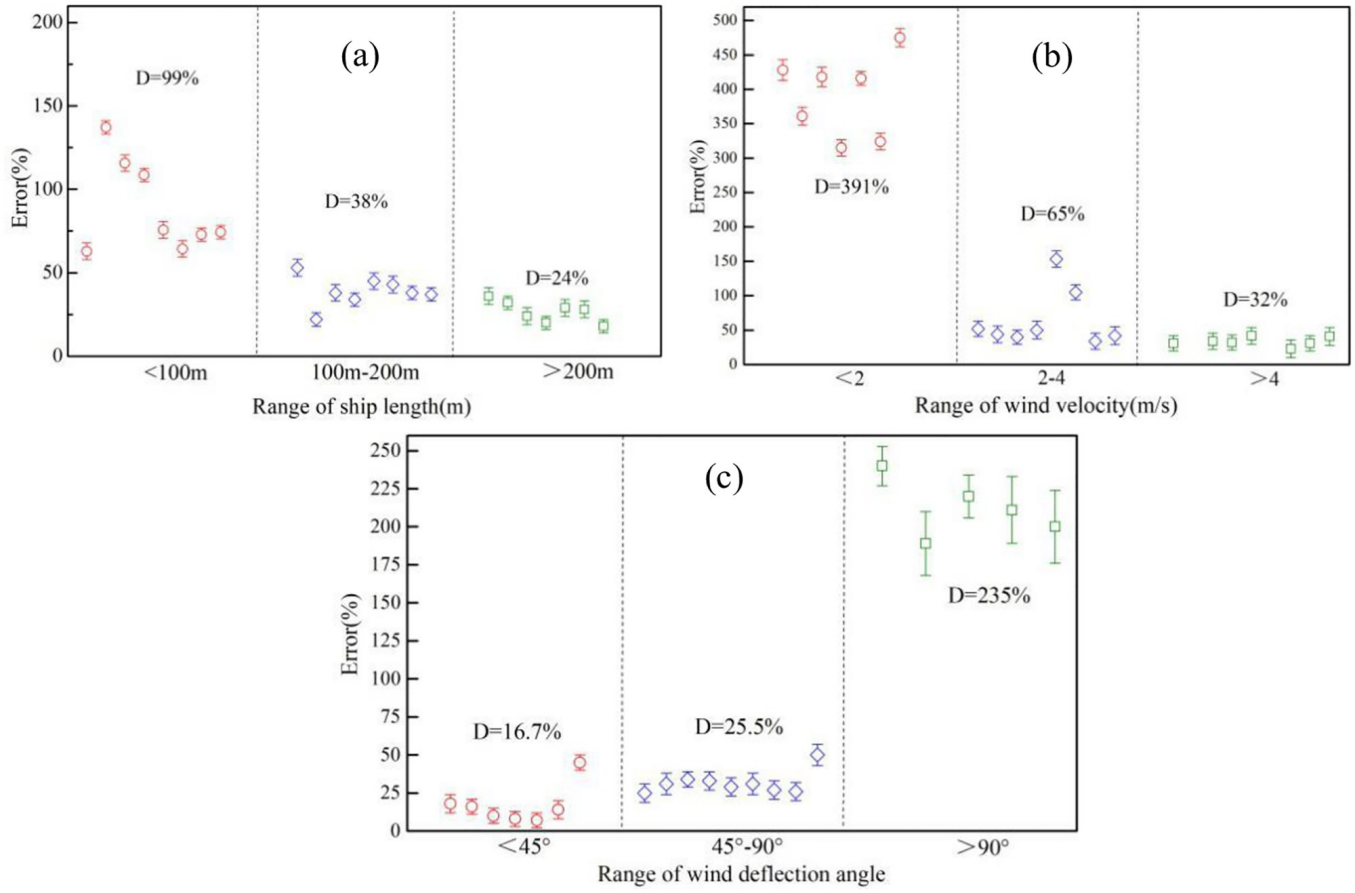
Influence of (a) ship size, (b) wind velocity and (c) wind deflection angle on the accuracy of model.

In the first emission source identification process, both exhaust emission prediction model and Gauss diffusion model were applied to calculate the theoretical pollutant concentration. As introduced in Section 2.3 the combined accuracy of the two models was defined as the difference between the real pollutant concentration detected by the sniffer system and the theoretical pollutant concentration. The accuracy of the exhaust emission prediction model is influenced by the ship parameters while the latter is dependent on the wind velocity and direction. Fig 9(a) shows the influence of ship length on model accuracy under similar wind velocity and direction. With the ship length below 100m, the average model error is as high as 99%, indicating that the pollutant emission factor is not fully applicable to small ships. When the ship length is higher than 200m, the average model error falls to 24%, demonstrating that the conventional prediction model needs to be further improved. With enough experimental data collected for a long time, a more precise ship exhaust prediction model as well as pollutant emission factor database can be established in the future.

**Fig 10:**
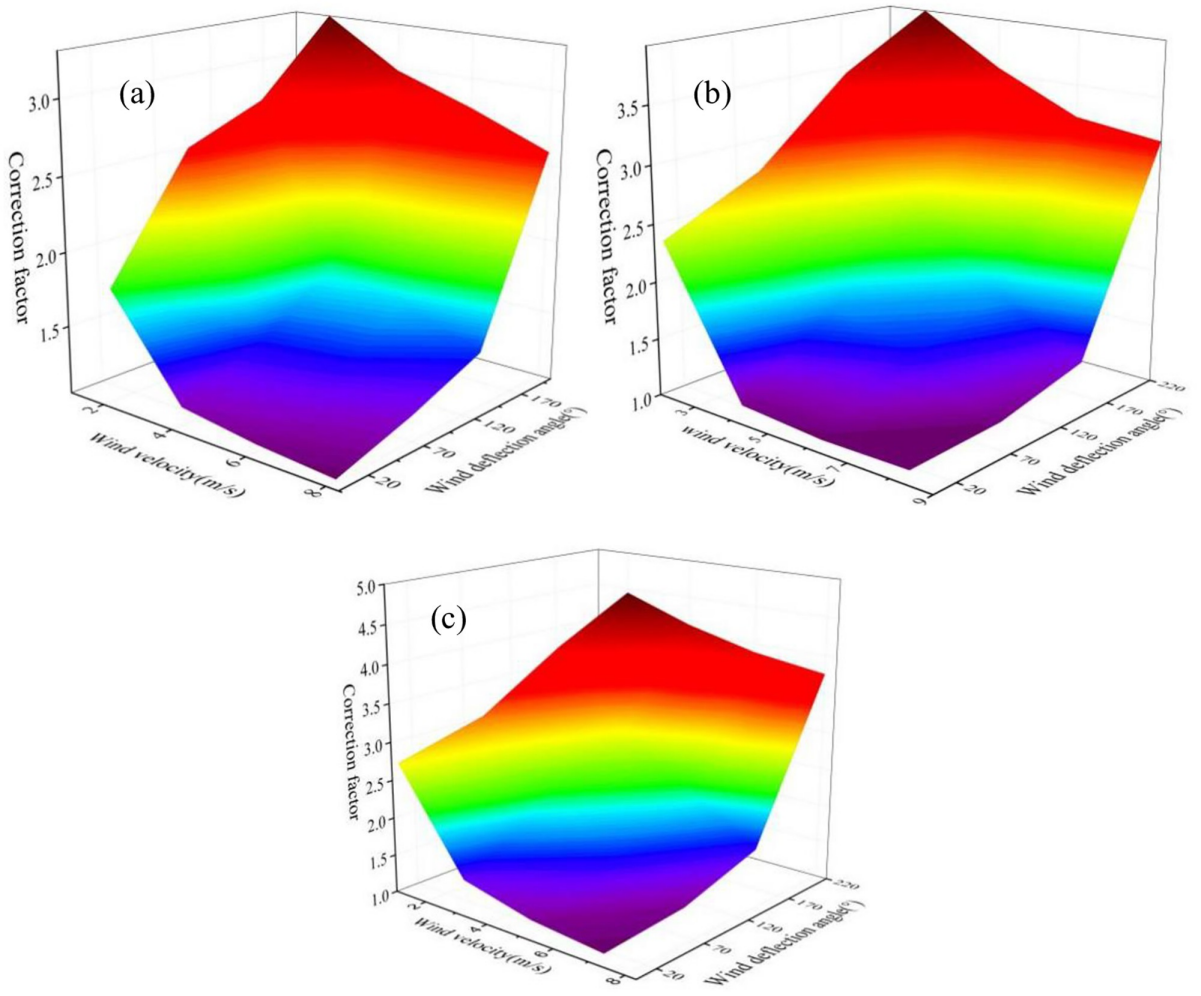
Map of correction factor under different wind velocity and deflection angle at the monitoring distance of (a) 300 m, (b) 500 m and (c) 800 m.

Fig 9(b) and 9(c) illustrates the influence of wind velocity and direction on accuracy of Gauss Diffusion model. It can be seen that with the decrease of the wind velocity, the model accuracy rise apparently. When the wind velocity is below 2 m/s, the average model error is as high as 391%. This may be due to the fact that the pollutant free diffusion velocity cannot be completely ignored in the Gauss Diffusion model when the wind velocity is low. It can be seen from S2 Fig in S1 File that in most cases the wind velocity is higher than 2m/s in the detection area. In contrast, the wind deflection angle exerts little effect on model accuracy when the monitoring site is in the downwind of the ship lane. However, the model error is much high when the wind deflection angle is over 90°, indicating that at least two sniffer station was demanded at two sides of the ship lane. A good configuration of the sniffer stations will further reduce the detection error and it is suggested that the angle between station to station line and the prevailing wind direction should be lower than 45°. Based on the results above, the map of model error can be obtained taking monitoring distance, wind velocity and direction as independent variables, and the results are shown in Fig 10. Considering the low chance of emergence of extreme weather condition with wind velocity lower than 2m/s, the screening criteria in the first emission source identification process can be set as that the ratio of theoretical NO_*x*_ concentration to the tested value should be in the range of 1/3 to 3. Ships in the “suspect list” that did not satisfy this criteria will be removed out.

### 3.4 Source trace accuracy

**Fig 11:**
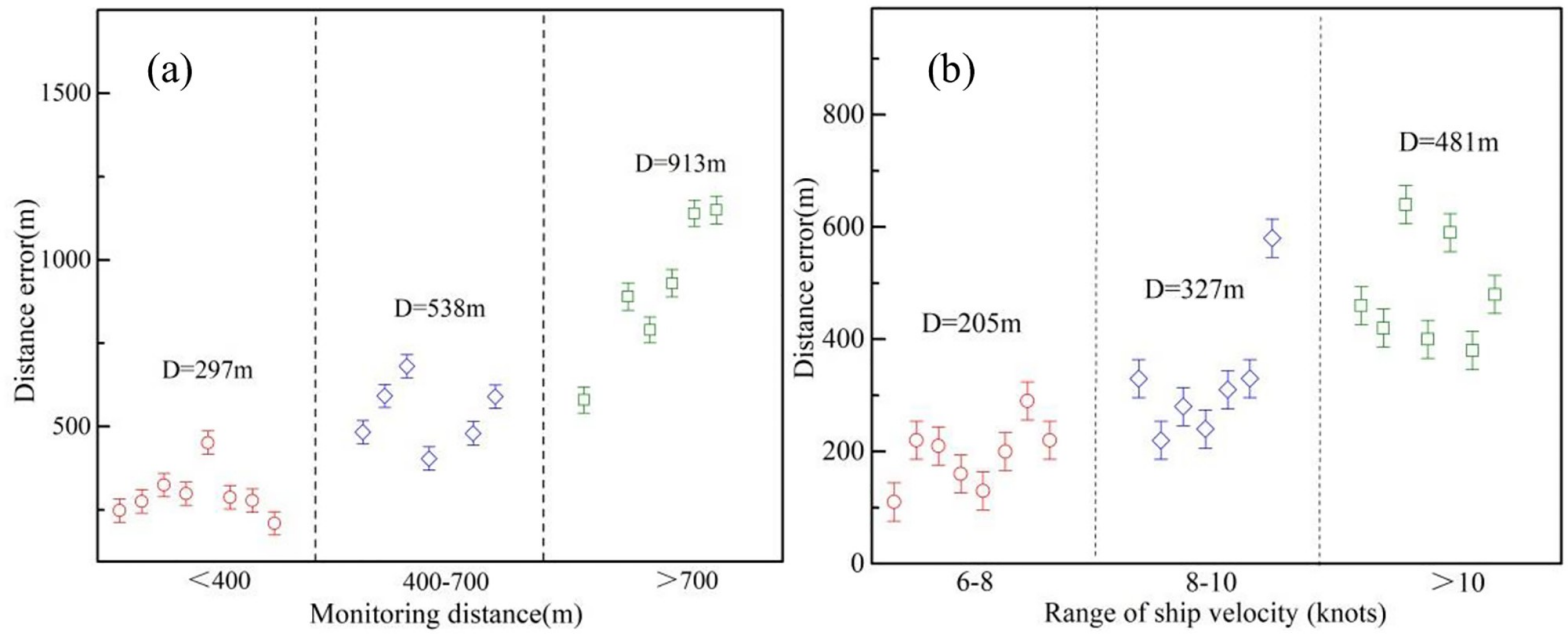
Influence of (a) monitoring distance and (b) ship velocity on the trace model accuracy.

In the second emission source identification process, the source trace model was applied to further filter the target ship in the “suspect list”. The distance between the theoretical source position and “special point” can be deemed as the index for the evaluation of the source trace accuracy. Based on analysis of experimental data, it can be concluded that the source trace accuracy is mainly dependent on ship speed and monitoring distance. Fig 11a illustrates the influence of the monitoring distance on the source trace accuracy. With the distance above 700 m, the average distance error can achieve over 900 m, indicating that if the two suspect ships pass through the monitoring site successively within a distance of 900 m, it is hard to tell which one is the target emission source. When the traffic is busy, the shortest gap between ships can be no more than 500 m, thus the monitor site should be placed close to the ship lane within the distance range of 400 m.

As shown in Fig 11b, the distance error was positively associated with ship speed. The pollutant diffusion speed in the source trace model was suggested as a fixed value obtained by a vector addition of ship and wind velocity. Actually the air friction in positive correlation with the velocity along the ship course will exert resistance effect on the velocity, leading to a considerable error to the final results. A considerable amount of vessels through the ship lane usually travel at a certain speed of 12 knots, indicating that the distance error can achieve around 500 m in most cases. A velocity correction factor can be used to reduce the effect of air friction and further improve the trace model accuracy.

### 3.5 Ship marking rate

**Fig 12:**
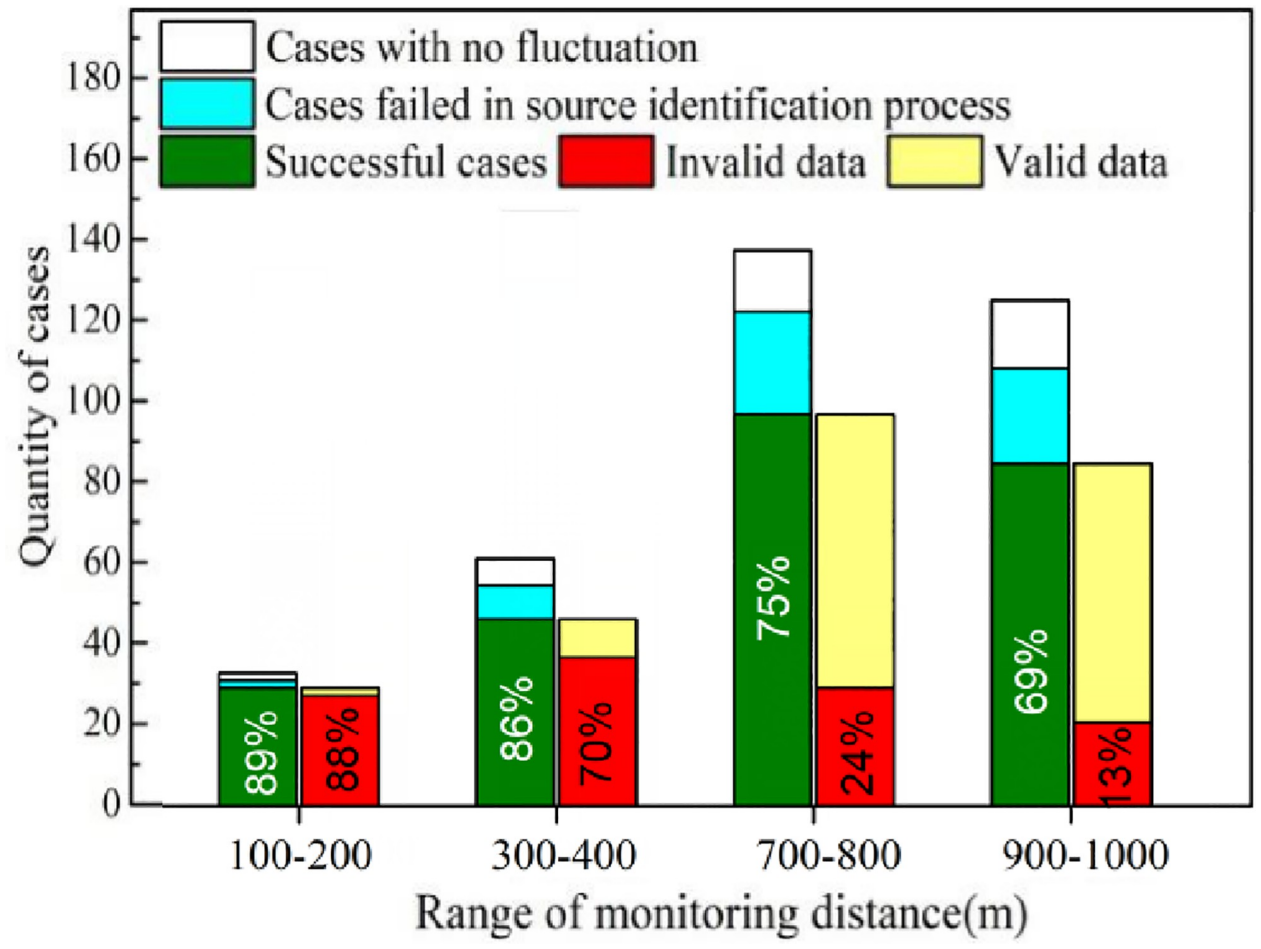
Influence of monitoring distance on ship marking rate and valid ship marking rate.

**Fig 13:**
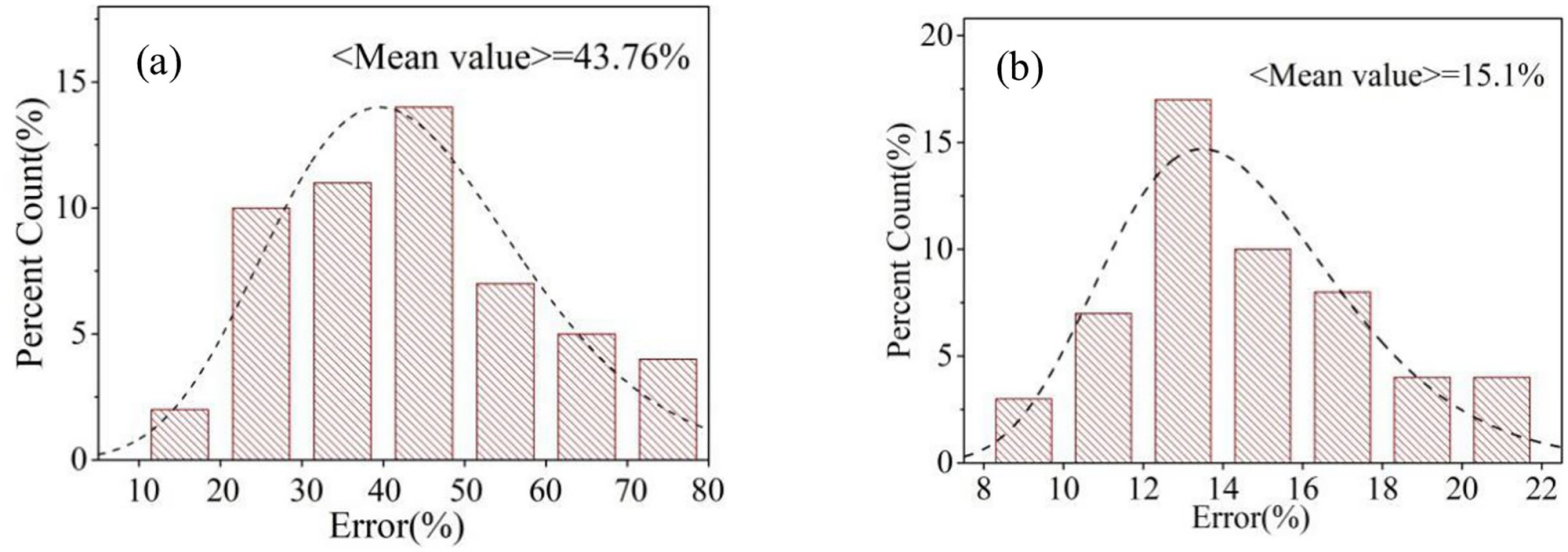
Error distribution of the results based on (a) conventional pollutant emission factor and (b) improved pollutant emission factor.

After the fluctuation generation, recognition and emission source identification, the target ship can be marked successfully. Thus the ship marking rate is a comprehensive performance index based on Fluctuation Generation Rate, Fluctuation Recognition Rate, Model Accuracy and Source Trace Accuracy. In our study, the only adjustable variable was monitoring distance. Thus long term experiment at different monitoring sites was performed and the average ship marking rate was obtained and illustrated in Fig 12. It can be seen that if the monitoring site is within 800 m off the ship lane, the ship marking rate can reach as high as 75%. However, not all the marked ship’s emission data are valid since the CO_2_ signal fluctuation may not emerge due to the relatively poor accuracy of CO_2_ sniffer equipment with the minimum unit of ppm. Fig 13 also shows the valid ship marking rate at different monitoring sites. It can be seen that with the monitoring distance longer than 700 m, only less than 24% of all marked ships’ exhaust aroused visible fluctuation of the CO_2_ signal and the oil sulfur content can be obtained through Eq 2. In addition to reducing the monitoring distance, another way to increase valid ship marking rate is using the detected NO_*x*_ data to estimate the CO_2_ concentration according to the emission factors summarized in S2 Table in S1 File. The equation is as follows:

$${\text{C}}_{\text{CO2}}{=\text{C}}_{\text{NOx}}\times \frac{{\text{E}}_{\text{CO2}}}{{\text{E}}_{\text{NOx}}}$$ (9)

Where C_CO2_ and C_NOx_ represent the concentration of CO_2_ and NO_*x*_, E_CO2_ and E_NOx_ represent the emission factor of CO_2_ and NO_*x*_. By comparing the calculated CO_2_ value with the actual CO_2_ concentration from the valid marked ship’s monitoring data, the accuracy of this method can be evaluated and the results are shown in Fig 13(a). It can be seen that the calculation accuracy is relatively low, indicating that emission factors in previous literature need to be further improved. According to the results, although the modified emission factors of CO_2_ and NO_*x*_ cannot be obtained, the ratio of two emission factors with more accuracy can be proposed, as listed in Table 2. Based on newly proposed factor ratio, the calculated CO_2_ value is more close to the actual value with error less than 15%, as shown in Fig 13(b). The final oil sulfur content will be a value with error range, such as 0.5%±0.08%. This is an alternative method when the monitoring site is far from the ship lane and the valid ship marking rate is very low.

**Table 2:** The improved emission factor ratio of CO_2_ to NO_*x*._

	Passenger ship	Tanker	Bulk cargo ship	Container ship	General cargo ship	Tugboat
Previous value	52	46	34	35	68	49
Improved value	43	68	44	27	85	43

### 3.6 Accuracy of the oil sulfur content

**Fig 14:**
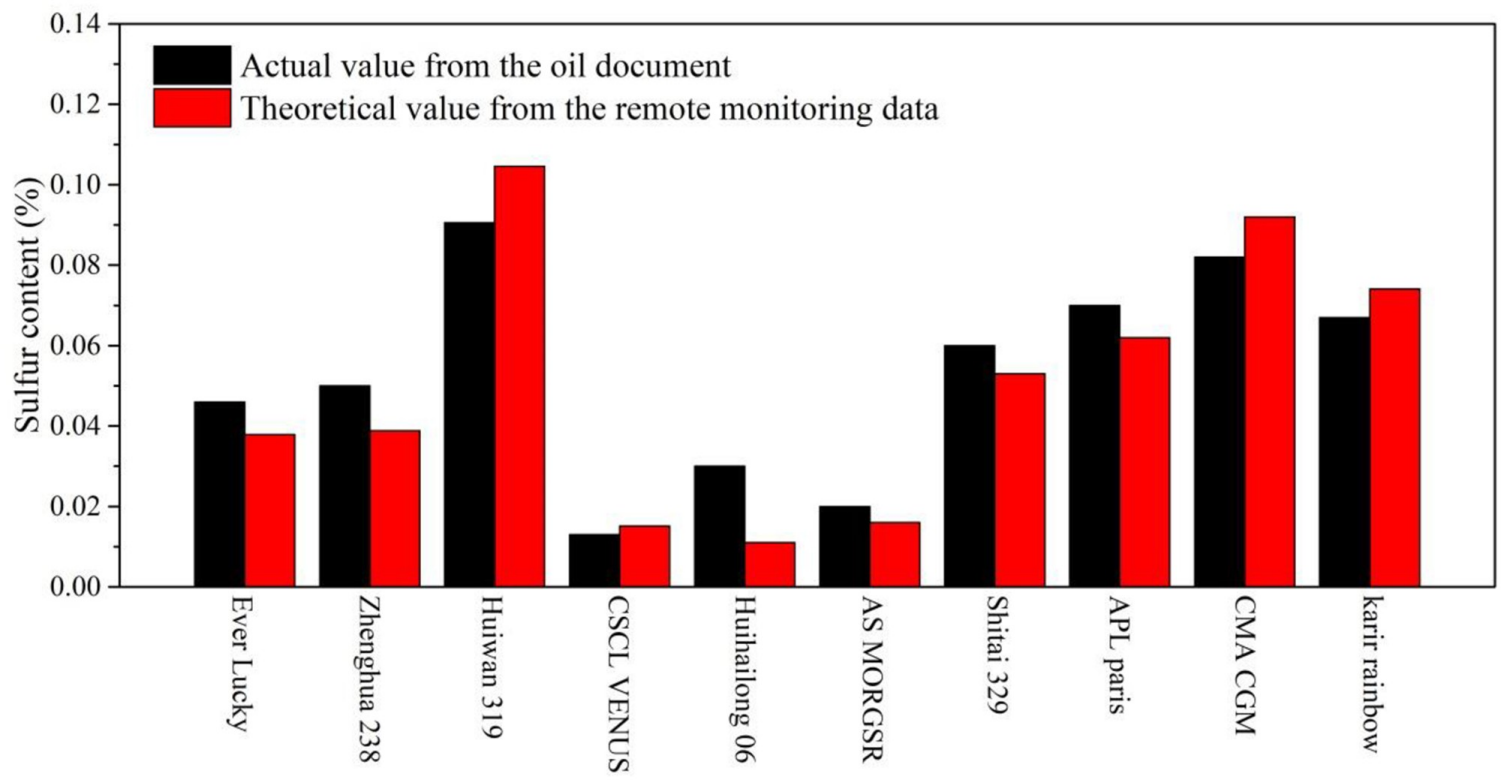
Comparison of real sulfur content and calculated sulfur content.

**Fig 15:**
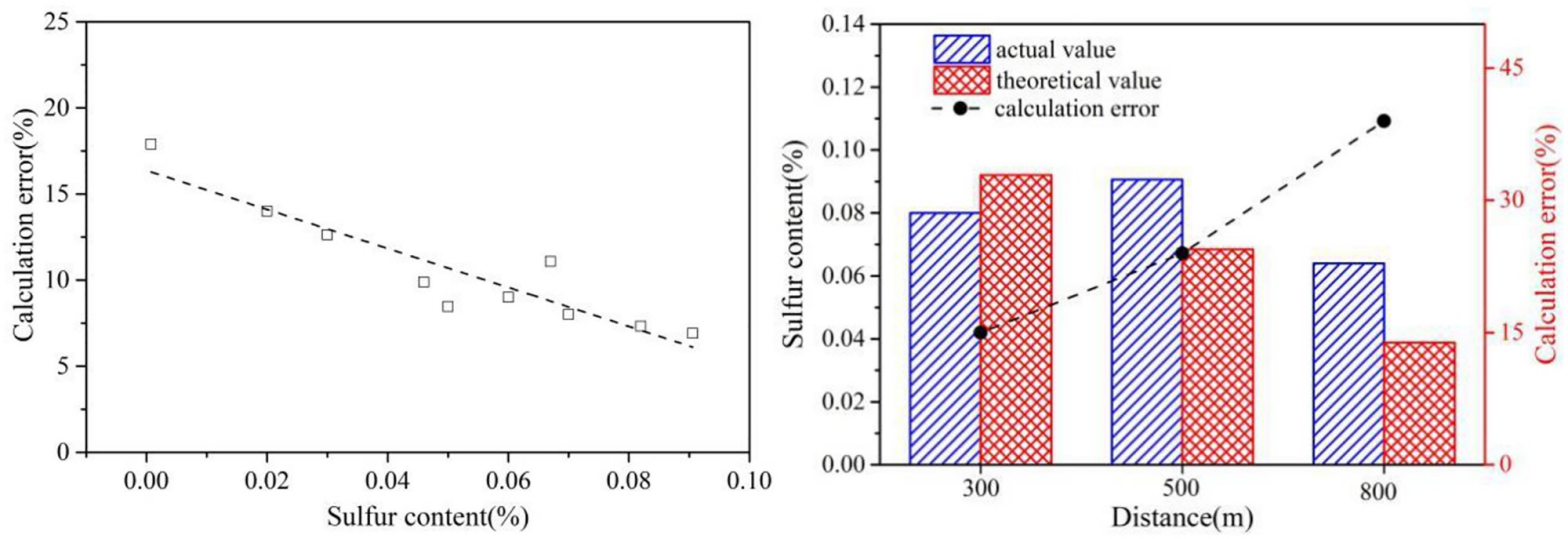
Influence of (a) sulfur content, (b) monitoring distance on the calculation error.

Fig 14 illustrates the difference between calculated sulfur content and real sulfur content obtained from the oil document provided by the marine bureau. As shown in Fig 15a, the error of detected oil sulfur content is in negative correlation with actual sulfur content. When the sulfur content is as low as 0.05%, the error could be higher than 25%. This may be due to the fact that the SO_2_ concentration detected by the sniffer station is lower than the SO_2_ sensor precision with poor sulfur content in the oil. Since the key monitoring objects are ships with heavy oil, the increased accuracy with sulfur content is favorable for the feasibility of the sniffer monitoring system. Similarly, It can be seen from Fig 15b that the long monitoring distance will also result in high calculation error due to high dilution, indicating that the sniffer station should be no more than 400m away from the ship lane in order to guarantee high calculation precision.

Based on the above results and discussion, it can be concluded that under complex and volatile marine meteorological environment, keeping the sniffer station in a close distance away from the ship lane is necessary to improve the efficiency, accuracy and feasibility of the monitoring system. However, it is not possible to place the sniffer station on the patrol boat since the patrol boat anchoring too close to the ship lane will disrupt the ship traffic. Also it is meaningless to apply shore-based sniffer system due to the fact that most ships near the port will shut down the main engine and the detected data cannot reflect the actual sulfur content. In order to realize the remote monitoring of ship emission with high efficiency and automation, the buoy based sniffer technique is a feasible way since the buoy locate near the ship lane far off shore with distance no more than 300 m, which will be intensely investigated in our future study.

## 4. Conclusion

In this study, a marine based sniffer technique was designed to perform remote monitoring of ship pollutant emission. A series of computational model were developed to automate the monitoring process. The feasibility of the monitoring system was detailed verified based on long term experiment and the influence of monitoring distance, ship parameters, wind velocity and direction on the efficiency and accuracy of the monitoring system were thoroughly investigated.

The ship marking rate is determined by the pollutant fluctuation generation rate and efficiency of emission source identification. The former is in negative correlation to monitoring distance as well as wind velocity and deflection angle while the later is dependent on the accuracy of exhaust emission prediction model, Gauss diffusion model and source trace model which are further influenced by ship size and velocity apart from the wind characteristics and monitoring distance. Monitoring distance is the only controllable factor and if the monitoring site is within 800 m off the ship lane, the ship marking rate can reach as high as 75%.

Despite high ship marking rate, the proportion of valid data is low due to the relatively poor accuracy of CO_2_ sniffer equipment with the minimum unit of ppm. In addition to further reducing the monitoring distance, a CO_2_ prediction model was proposed to increase data valid rate to 100% and the emission factor in previous database was further improved based on the experimental results. Accuracy of the oil sulfur content is in positively relation with actual sulfur content in the oil, which is favorable for the feasibility of the sniffer monitoring system considering the Key monitoring objects. Monitoring distance is another factor and sniffer station should be no more than 400m away from the ship lane in order to guarantee high calculation precision.

Based on the results and discussion, the principle of and site selection of sniffer system were proposed. and a novel sniffer monitoring system with two buoy based sniffing stations placed close to each side of the ship lane far off shores was proposed to realize the remote monitoring of ship emissions.

The obtained final result is the sulfur content of the oil used by the ship. If the result is higher than emission limit of 0.5%, it means the detected ship is in violation of emission rules set by IMO and should be punished.

However, though the computational models in this work were modified to further increase the precision based on the collected date, the uncertainties of the results still exist since the obtained fuel sulfur content is influenced by a combination of factors such as wind speed, wind direction, object ship parameters and monitoring distance. In this study the uncertainty of the result cannot be estimated precisely since the actual sample size obtained from the maritime sector are not enough. An empirical equation will be proposed based on the multivariate analysis in future study in order to further estimate the uncertainties of the result.

## Supporting information

S1 File (DOCX)
