# Supplementary material for: Investigation of sniffer technique on remote measurement of ship emissions: A case study in Shanghai, China
Source: PLoS One. 2022 Sep 16;17(9):e0274236. doi: 10.1371/journal.pone.0274236 (PMC9481039; doi:10.1371/journal.pone.0274236)
Supplement: S1 File — (DOCX) [file pone.0274236.s001.docx]

Table S1 Load factor of auxiliary engine of different ship at different operating state

| Ship type | Load factor | | | | | |
| --- | --- | --- | --- | --- | --- | --- |
|  | Sailing | Slowing down | | Motorizing | | Anchoring |
| bulk cargo ship | 0.17 | | 0.27 | | 0.45 | 0.22 |
| container ship | 0.13 | | 0.25 | | 0.50 | 0.17 |
| Tourist boats | 0.80 | | 0.80 | | 0.80 | 0.64 |
| General cargo ship | 0.17 | | 0.27 | | 0.45 | 0.22 |
| Tugboat | 0.17 | | 0.27 | | 0.45 | 0.22 |
| Refrigerator ship | 0.20 | | 0.34 | | 0.67 | 0.34 |
| Tankship | 0.13 | | 0.27 | | 0.45 | 0.67 |

Table S2 Exhaust factor of typical type of ship at different operating state

| State | Pollutant | Passenger ship | tanker | bulk cargo ship | Container ship | General cargo ship | Tugboat |
| --- | --- | --- | --- | --- | --- | --- | --- |
| Sailing | NO*_x_* | 13.2 | 14.9 | 17.9 | 17.5 | 11.1 | 13.7 |
|  | SO_2_ | 11.7 | 11.7 | 10.6 | 10.7 | 12.9 | 10.8 |
|  | CO_2_ | 696 | 689 | 624 | 624 | 757 | 673 |
|  | HC | 0.5 | 0.5 | 0.6 | 0.6 | 0.4 | 0.4 |
|  | PM | 2.3 | 2.3 | 2.3 | 2.3 | 2.2 | 2.3 |
| Entering or leaving port | NO*_x_* | 13.2 | 14.9 | 17.9 | 17.5 | 11.1 | 13.7 |
|  | SO_2_ | 11.7 | 11.7 | 10.6 | 10.7 | 12.9 | 10.8 |
|  | CO_2_ | 696 | 689 | 624 | 624 | 757 | 673 |
|  | HC | 0.5 | 0.5 | 0.6 | 0.6 | 0.4 | 0.4 |
|  | PM | 2.3 | 2.3 | 2.3 | 2.3 | 2.2 | 2.3 |
| Staying in port | NO*_x_* | 11.6 | 12.1 | 13.8 | 13.7 | 11.8 | 11.8 |
|  | SO_2_ | 12.6 | 12.8 | 12.0 | 12.1 | 12.0 | 12.0 |
|  | CO_2_ | 750 | 754 | 706 | 710 | 761 | 734 |
|  | HC | 1.0 | 1.4 | 1.0 | 1.0 | 0.7 | 1.0 |
|  | PM | 1.8 | 2.2 | 1.5 | 1.5 | 1.4 | 1.8 |

Table S3 Input parameter,intermediate calculation parameter and results in one case

| Longitude(°) | 114 |
| --- | --- |
| Latitude(°) | 31 |
| Wind velocity(m/s) | 3 |
| Power_ship(kW) | 3300 |
| Angle of wind direction(rad) | 2.8 |
| Angle of ship course direction(rad) | 5.1 |
| Angle of the line vector from ship to the monitoring site(rad) | 4.18 |
| Distance between the ship to the monitoring site | 576 |
| Type_ship | Container ship |
| Velocity of ship(Knots) | 11.8 |
| L_F_ | 0.75 |
| Emission factor of NOx | 17.5 |
| calculated NOx concentration in the pipe(g/s) | 5.1 |
| Day number (from 1 to 365) | 108 |
| Angle of the day(rad) | 1.86 |
| Wind deflection angle(rad) | 1.37 |
| Angle of sun inclination(rad) | 0.190 |
| Angle of sun height(rad) | 2.7 |
| Corresponding angle of time(rad) | 0.1673 |
| Distance traveled in the last 20 minute(meters) | 6065 |
| Radiation level of the sun | 0 |
| Atmospheric Stability level | 4 |
| $\text{γ}_{\text{1}}$ | 0.826 |
| $\text{α}_{\text{2}}$ | 0.929 |
| $\text{γ}_{\text{1}}$ | 0.105 |
| $\text{α}_{\text{2}}$ | 0.111 |
| z | 10.6 |
| Parameter diffusion of z | 17.3 |
| Height of the ship pipe above the ground monitoring equipment(meters) | 9.5 |
| Theoretical NOx concentration(ppm) | 32.3 |
| Detected NOx concentration(ppm) | 27.6 |
| Distance between the line vector between the calculated emission source site to the monitoring site(rad) | 639 |
| Angle of the line vector between the calculated emission source site to the monitoring site(rad) | 4.53 |
| Detected FSC(%) | 0.32 |
| exceed emission standard or not | No |


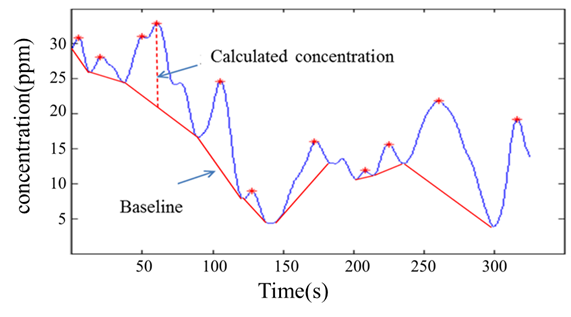


Figure S1 Schematic of determination of baseline and peak value


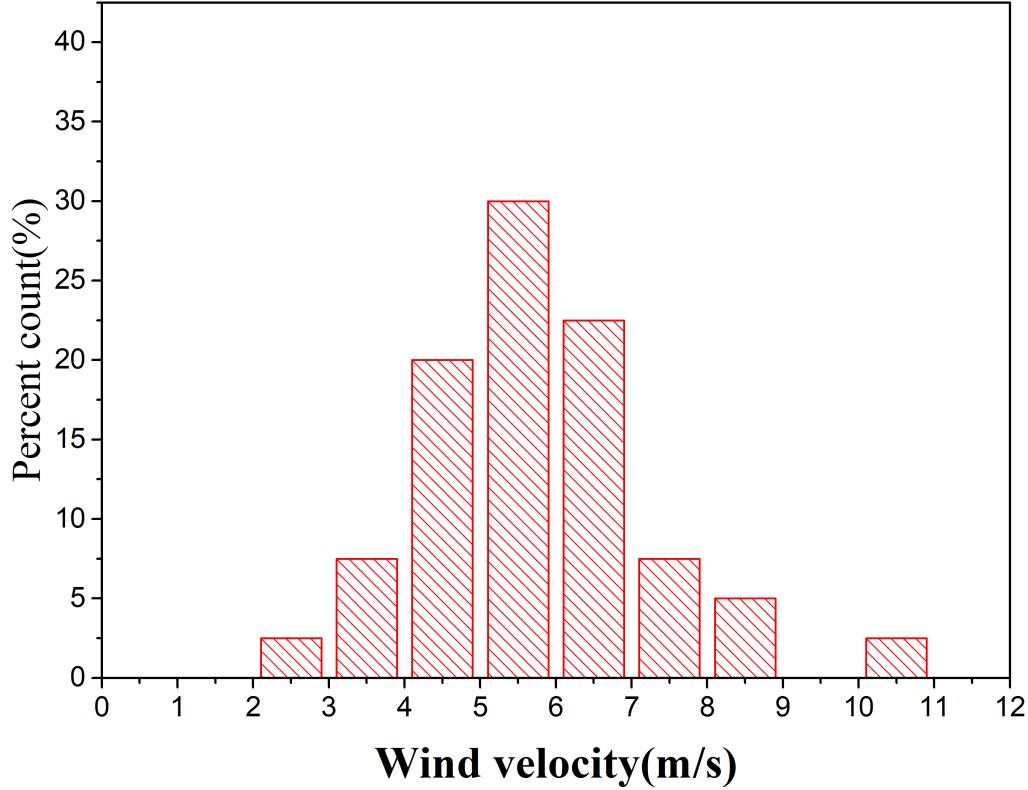


Figure S2 Statistics of wind speed data in the detection area
